# Prevalence, Awareness, Treatment and Control of Diabetes in India From the Countrywide National NCD Monitoring Survey

**DOI:** 10.3389/fpubh.2022.748157

**Published:** 2022-03-14

**Authors:** Prashant Mathur, Sravya Leburu, Vaitheeswaran Kulothungan

**Affiliations:** Indian Council of Medical Research—National Centre for Disease Informatics and Research, Bengaluru, India

**Keywords:** awareness, control, diabetes mellitus, India, prevalence, treatment, surveillance

## Abstract

**Background:**

To determine the prevalence, awareness, treatment and control of diabetes mellitus (DM) and associated factors amongst adults (18–69 years) in India from the National Noncommunicable Disease Monitoring Survey (NNMS).

**Methods:**

NNMS was a comprehensive, cross-sectional survey conducted in 2017–18 on a national sample of 12,000 households in 600 primary sampling units. In every household, one eligible adult aged 18–69 years were selected. Information on NCD risk factors and their health-seeking behaviors were collected. Anthropometric measurements, blood pressure and fasting capillary blood glucose were measured. DM was defined as fasting blood glucose (FBG) ≥126 mg/dl including those on medication. Awareness, treatment, and control of DM were defined as adults previously diagnosed with DM by a doctor, on prescribed medication for DM, and FBG <126 mg/dl, respectively. The weighted data are presented as mean and proportions with 95% CI. We applied the Student *t*-test for continuous variables, Pearson's chi-square test for categorical variables and multivariate regression to determine the odds ratio. For statistical significance, a *p*-value < 0.05 was considered.

**Results:**

Prevalence of DM and impaired fasting blood glucose (IFG) in India was 9.3% and 24.5% respectively. Among those with DM, 45.8% were aware, 36.1% were on treatment and 15.7% had it under control. More than three-fourths of adults approached the allopathic practitioners for consultation (84.0%) and treatment (78.8%) for diabetes. Older adults were associated with an increased risk for DM [OR 8.89 (95% CI 6.66–11.87) and were 16 times more aware of DM. Better awareness, treatment and control levels were seen among adults with raised blood pressure and raised cholesterol.

**Conclusions:**

The prevalence of DM and IFG is high among adults, while the levels of awareness, treatment and control are still low in India, and this varied notably between the age groups. Multifaceted approaches that include improved awareness, adherence to treatment, better preventive and counseling services are crucial to halt diabetes in India. Also, expanding traditional systems of medicine (Ayurveda, Yoga, Naturopathy, Unani, Siddha, and Homeopathy [AYUSH]) into diabetes prevention and control practices open solutions to manage this crisis.

## Introduction

Diabetes is a rapidly growing health challenge and potential epidemic across the low-and-middle-income countries like India (1). It is projected that by 2025 the number of cases with diabetes in India would be 69.9 million with a vast majority still undiagnosed (1, 2). This is primarily driven by dietary transitions and insufficient or lack of physical activity altering the physiological milieu leading to overweight or obesity and diabetes (1, 2). Care for chronic diseases like diabetes poses challenges characterized by the need for sustained compliance to treatment, prevention or management of associated complications (3). This requires the continuous engagement of health systems in the continuum of care at all stages (3). Diabetes care requires coordination across all tiers of health care systems. Most importantly co-driven by the patient's knowledge, attitudes and perceptions toward awareness, treatment and adherence to the recommendations (3, 4).

The Noncommunicable Disease (NCD) Monitoring Framework targets (10) and indicators (21) set by the Ministry of Health and Family Welfare, Government of India adapted from the Global NCD framework (World Health Organization), calls for a need to halt the rise in diabetes and prevent premature deaths from NCDs by 25% by 2025 (5, 6). Such targets can be met only with effective strategies at multisectoral levels (7). However, an important limitation and quandary for policymakers are that majority of the population might be unaware of their diabetes status and are not adherent to advice (3). Robust empirical data on diabetes prevalence, awareness, treatment, control and adherence is needed to comprehend the impact of initiatives taken to halt the growing burden of diabetes, response of health systems and health-seeking behaviors amongst the population (1). Understanding where diabetics are lost in the care cascade is essential for targeted health interventions. Also, to monitor progress in health system performance for diabetes management over time (8).

In this paper, we present the results on diabetes care cascade among those aged 18–69 years from the large national comprehensive survey, the National NCD Monitoring Survey (NNMS). Additionally, the paper also presents results on the availability of services for diabetes care amongst surveyed public health facilities across the country. The NNMS was undertaken to collect much-needed quality data specific to NCD risk factors in adults and adolescents, health-seeking behaviors and health system responses to NCDs in India (9, 10).

## Materials and Methods

### Sampling Design

The NNMS was a multi-centric, cross-sectional survey done in 2017–18, that addressed NCD specific components at the population level among adults: 18–69 years and adolescents: 15–17 years residing in urban and rural areas; and at the health facility level. The survey was coordinated and implemented by the Indian Council of Medical Research (ICMR)—National Center for Disease Informatics and Research (NCDIR), Bengaluru (9, 10).

The survey followed a multistage cluster sampling design by dividing the country into 10 contiguous zones that approximated 60 clusters and 600 primary sampling (300 rural and 300 urban). The study population was divided into four subgroups/strata urban/rural and men/women (2 x 2). The sample size for adults aged 18–69 years was computed using 9% estimated prevalence of obesity, 15% relative precision, 95% confidence interval, 15% Non-response rate and design effect of 1.5. Since the adolescents (15–17 years) were to be enrolled from the same households, the sample was enlarged to 12,000 households and this was equally allocated to both urban and rural areas (6,000 households each). Twenty households were selected in every PSU to sum up to 12,000 households to represent a national sample. One eligible adult aged 18–69 years from every household was selected by the KISH method thus, totalling a sample of 12,000 adults. For the health facility survey, one each of public primary, community health centers, district hospital and primary private hospitals within and, near the PSUs were included in the survey sample (9–11).

The survey was approved by the ICMR-NCDIR institutional ethics committee (IEC) and the respective survey implementing agencies IECs. The survey obtained all the necessary support and concurrence from local bodies for its implementation. All selected study participants were briefed about the visit and the purpose of the survey. Following their voluntary acceptance to participate, written informed consent was obtained.

### Data Collection and Laboratory Methods

Survey data were collected electronically in personal digital assistants through globally standardized questionnaires [WHO-STEPwise approach to noncommunicable disease risk factor surveillance (WHO-STEPS), Integrated disease surveillance project (IDSP)-NCD risk factor survey, and Global Adult Tobacco Survey-India (GATS)] administered by well-trained interviewers in English and eleven local languages through face-to-face interviews at the household (9, 10). Physical measurements of height (SECA 213 portable stadiometer), weight (SECA 803 digital weighing scale), waist circumference (SECA, 201 measuring or tension tape), blood pressure (OMRON HEM−7120 automatic blood pressure machine) were also made at the household level by trained and certified technicians using international standard procedures recommended by WHO-STEPS (9, 10). All measures of privacy and confidentiality were followed to limit any possible bias during data collection. Biochemical testing of fasting blood glucose (FBG) was done as a camp-based approach among consenting adults. During the household interviews, information on socio-demographic characteristics (e.g., education level, occupation, housing type etc.) and risk factors like tobacco use, alcohol consumption, dietary factors (intake of fruits and vegetable intake, dietary salt), levels of physical activity (moderate and vigorous physical activity at workplace or home, during travel and leisure) using Global Physical Activity Questionnaire were collected, including questions on previous diagnosis, treatment of diabetes, hypertension, raised blood cholesterol and cardiovascular or cerebrovascular accidents (9, 10). The study questionnaires for health-seeking behaviors also included questions on consultation and treatment-seeking behaviors of adults from practitioners of allopathy or alternate system of medicine including those who practised Ayurveda, Yoga and Naturopathy, Unani, Siddha and Homeopathy (AYUSH) (9).

All eligible participants were given appointment slips a day before the camp along with instructions for fasting. One place in the PSU was identified based on operational feasibility for setting up the camp. All participants were called to the camp facility early in the morning in an overnight fasting state (≥ 8 h). The date and time of their last meal were noted in the camp activity sheet. On confirmation of fasting status and under aseptic conditions the capillary fasting blood glucose estimation was done using Glucometer *(Gluco spark, Sensa core, Telangana, India)* by teams well-trained in all survey procedures including laboratory procedures, sample handling and waste disposal (9, 10).

### Definitions and Statistical Analysis

According to the WHO diagnostic criteria, prevalence of diabetes mellitus (DM) was defined as FBG ≥126 mg/dl or self-reported history of diabetes (i.e., if they have ever been diagnosed with DM as told by a doctor or health professional) and impaired fasting blood glucose (IFG) was defined as FBG 100–125 mg/dl (12). Adults who self-reported were considered as previously diagnosed/aware and those who had raised FBG levels ≥126 mg/dL on testing during the survey but did not self-report were classified under newly-diagnosed cases of DM. Treatment was defined as the use of anti-diabetic medications (oral hypoglycaemic drugs or insulin) for DM on any one day in the last 2 weeks before the survey. Control was defined as treatment (oral medication or insulin) of DM associated with FBG <126 mg/dl when measured for FBG in the survey (9).

Standard definitions were used for estimating all behavioral and biological indicators (tobacco use, alcohol use, diet, physical activity, BMI, central obesity, raised blood pressure and raised cholesterol). Current tobacco and alcohol use was defined as the use of any form of tobacco (smoked or smokeless) and consumption of alcohol in the last 12 months preceding the survey. Insufficient physical activity in adults was defined as the proportion of adults aged 18–69 years who spent <150 min of moderate-intensity physical activity per week OR <75 min of vigorous-intensity physical activity per week OR an equivalent combination of moderate-and-vigorous intensity physical activity accumulating <600 MET-min per week. BMI was categorized using WHO criteria: underweight: <18.5 Kg/m^2^, Normal: 18.5–24.9 Kg/m^2^, Overweight: 25.0–29.9 Kg/m^2^ and obesity: ≥30.0 Kg/m^2^. Central obesity was defined as those with a waist circumference of ≥90 cm in males and ≥80 cm in females (as per South Asia Pacific Guidelines). Raised blood pressure in adults aged between 18 and 69 years with a systolic blood pressure ≥140 mmHg and/or diastolic blood pressure ≥90 mmHg including those on medication for raised blood pressure. Raised cholesterol was defined as all adults (18–69 years) who reported being diagnosed as having raised blood cholesterol either by a doctor or health worker.

The data collected was cleaned using *IBM Statistical Package for the Social Sciences (SPSS) for Windows version 22.0*. The cleaned data were weighted and analyzed in *STATA 14.1* using complex survey analysis. The survey response rates are provided as weighted numbers and proportions. The weighted results have been presented in descriptive statistics as mean and proportions with 95% confidence interval (CI). The association of variables with diabetes were assessed by the *Student t-test* for continuous variables and the *Pearson's chi-square test* for categorical variables. We performed the logistic regression analysis to determine the risk factors using odds ratio (OR) estimates with 95% CI. A multivariate regression analysis was done with a *p-*value < 0.05 for statistical significance.

## Results

**Figure 1:**
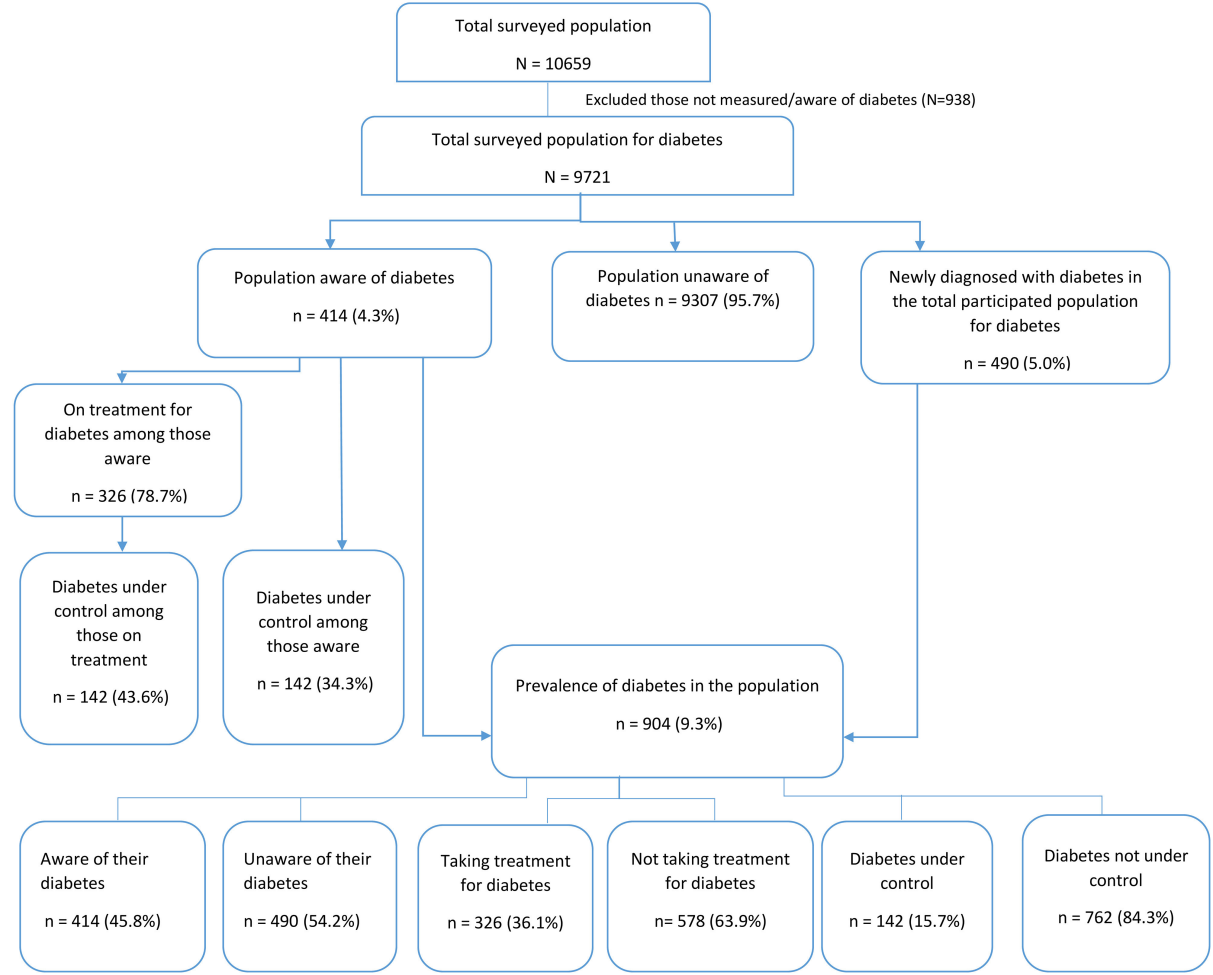
Survey response rates of diabetes care cascade among adults (18–69 years) in India.

A total of 9,721 adults were surveyed out of which 904 were found to have diabetes based on their FBG measurement and self-reported history of diabetes. Out of these, only 414 were aware of their diabetes status, 326 were under treatment for diabetes and 142 were under control as defined as fasting blood glucose <126 mg/dl (Figure 1).

The mean FBG among 18–69 years was 96.7 mg/dl, this was higher in the older age group 50–69 years (107.47 mg/dl) and among urban adults (101.57 mg/dl) (Supplementary Table S1).

Overall, 66.2% of adults were normoglycemic and 24.5% had IFG of 100–125 mg/dl. A nearly equal proportion of adults were newly-diagnosed (5.0%) and previously-diagnosed (4.3%) with diabetes. The highest proportion of adults with either IFG, newly-diagnosed or previously-diagnosed diabetes was aged 50–69 years, urban residents, and had metabolic risk factors (overweight, obesity, central obesity, hypertension and raised cholesterol). These findings were statistically significant (*p* < 0.001) (Table 1).

**Table 1:** Distribution (Percentage) of socio-demographic and risk factor profile of Indian adult population (18–69 years) by fasting blood glucose categories.

**Variables**	***n* (weighted)**	**Normal fasting blood glucose**			**Impaired fasting blood glucose**			**Newly-diagnosed diabetes**			**Previously-diagnosed diabetes**		
		***n* [%]**	**95% CI**	p^*^value	***n* [%]**	**95% CI**	***p* value**	***n* [%]**	**95% CI**	***p* value**	***n* [%]**	**95% CI**	***p* value**
**Overall (18–69 years)**	9,721	6,438 [66.2]	(63.6–68.8)		2,379 [24.5]	(22.3–26.7)		490 [5.0]	(4.2–6.0)		414 [4.3]	(3.7–5.0)	
**Residence**													
Urban	3,137	1,791 [57.1]	(52.1–62.0)	**<0.001**	896 [28.6]	(24.6–32.8)	**<0.001**	192 [6.1]	(4.9–7.7)	**0.001**	258 [8.2]	(6.9–9.7)	**<0.001**
Rural	6,584	4,647 [70.6]	(67.6–73.4)		1,483 [22.5]	(20.1–25.2)		298 [4.5]	(3.5–5.7)		156 [2.4]	(1.9–3.0)	
**Sex**													
Men	5,036	3,509 [69.7]	(67.0–72.2)	**<0.001**	1,100 [21.8]	(19.6–24.3)	**<0.001**	209 [4.2]	(3.4–5.1)	**<0.001**	218 [4.3]	(3.5–5.4)	0.723
Women	4,685	2,929 [62.5]	(59.2–65.7)		1,279 [27.3]	(24.7–30.0)		281 [6.0]	(4.8–7.4)		196 [4.2]	(3.5–5.0)	
**Age groups**													
18–29 years	2,853	2,264 [79.4]	(76.1–82.2)	**<0.001**	525 [18.4]	(15.8–21.3)	**<0.001**	50 [1.8]	(1.2–2.6)	**<0.001**	14 [0.5]	(0.2–1.1)	**<0.001**
30–49 years	4,681	3,095 [66.1]	(63.0–69.1)		1,222 [26.1]	(23.5–28.9)		243 [5.2]	(4.2–6.4)		121 [2.6]	(2.0–3.4)	
50–69 years	2,187	1,079 [49.3]	(45.6–53.0)		632 [28.9]	(25.9–32.1)		197 [9.0]	(7.3–11.1)		279 [12.8]	(10.9–14.9)	
**Education status**													
Received education	6,847	4,547 [66.4]	(63.5–69.2)	0.561	1,651 [24.1]	(21.8–26.5)	0.194	330 [4.8]	(4.0–5.8)	0.124	319 [4.7]	(3.9–5.5)	**0.003**
No education	2,874	1,891 [65.8]	(62.2–69.2)		728 [25.3]	(22.4–28.5)		160 [5.6]	(4.3–7.1)		95 [3.3]	(2.5–4.3)	
**Behavioral risk factors**													
**Current tobacco use (any form)**													
No	6,513	4,142 [63.6]	(60.6–66.5)	**<0.001**	1,696 [26.0]	(23.7–28.6)	**<0.001**	350 [5.4]	(4.4–6.5)	**0.032**	325 [5.0]	(4.3–5.8)	**<0.001**
Yes	3,208	2,296 [71.6]	(68.4–74.6)		683 [21.3]	(18.7–24.1)		140 [4.4]	(3.3–5.7)		89 [2.8]	(2.1–3.7)	
**Current alcohol use**													
No	8,164	5,335 [65.3]	(62.5–68.1)	**<0.001**	2,056 [25.2]	(23.0–27.5)	**<0.001**	419 [5.1]	(4.2–6.2)	0.347	354 [4.3]	(3.7–5.1)	0.377
Yes	1,557	1,103 [70.8]	(66.8–74.6)		323 [20.7]	(17.5–24.3)		71 [4.6]	(3.2–6.5)		60 [3.9]	(2.6–5.5)	
**Physical activity**													
Insufficient	4,002	2,458 [61.4]	(58.1–64.6)	**<0.001**	1,081 [27.0]	(24.4–29.8)	**<0.001**	222 [5.5]	(4.5–6.8)	0.056	241 [6.0]	(5.0–7.2)	**<0.001**
Sufficient	5,719	3,980 [69.6]	(66.7–72.3)		1,298 [22.7]	(20.4–25.2)		268 [4.7]	(3.8–5.8)		173 [3.0]	(2.4–3.8)	
**Metabolic risk factors**													
**BMI categories**													
Normal	5,198	3,566 [68.6]	(65.6–71.4)	**<0.001**	1,259 [24.2]	(21.8–26.8)	**<0.001**	222 [4.3]	(3.5–5.2)	**<0.001**	151 [2.9]	(2.3–3.7)	**<0.001**
Underweight	1,863	1,455 [78.1]	(74.8–81.1)		326 [17.5]	(14.8–20.6)		59 [3.2]	(2.3–4.4)		23 [1.2]	(0.6–2.3)	
Overweight	1,895	1,040 [54.9]	(50.5–59.1)		544 [28.7]	(25.2–32.6)		145 [7.7]	(6.0–9.7)		166 [8.8]	(7.4–10.3)	
Obesity	590	257 [43.6]	(38.2–49.2)		212 [35.9]	(30.2–42.0)		59 [10.0]	(6.8–14.4)		62 [10.5]	(8.0–13.7)	
**Central obesity**													
No	6,492	4,735 [72.9]	(70.3–75.4)	**<0.001**	1,391 [21.4]	(19.2–23.8)	**<0.001**	244 [3.8]	(3.1–4.6)	**<0.001**	122 [1.9]	(1.5–2.4)	**<0.001**
Yes	3,071	1,589 [51.7]	(47.9–55.5)		960 [31.3]	(28.2–34.5)		240 [7.8]	(6.2–9.8)		282 [9.2]	(8.0–10.5)	
**Raised blood pressure**													
No	6,917	4,822 [69.7]	(67.0–72.3)	**<0.001**	1,644 [23.8]	(21.5–26.2)	**0.011**	295 [4.3]	(3.5–5.2)	**<0.001**	156 [2.3]	(1.8–2.9)	**<0.001**
Yes	2,783	1,600 [57.5]	(53.9–61.0)		730 [26.2]	(23.5–29.2)		195 [7.0]	(5.6–8.7)		258 [9.3]	(7.9–10.9)	
**Reported raised cholesterol**													
No	9,592	6,411 [66.8]	(64.2–69.4)	**<0.001**	2,334 [24.3]	(22.2–26.6)	0.006	480 [5.0]	(4.2–5.9)	0.156	367 [3.8]	(3.3–4.5)	**<0.001**
Yes	129	27 [20.9]	(13.2–31.2)		45 [34.9]	(25.2–46.1)		10 [7.8]	(3.7–16.0)		47 [36.4]	(26.1–48.0)	

* *Chi-square test. P-value < 0.05 is considered statistically significant*.

**Figure 2:**
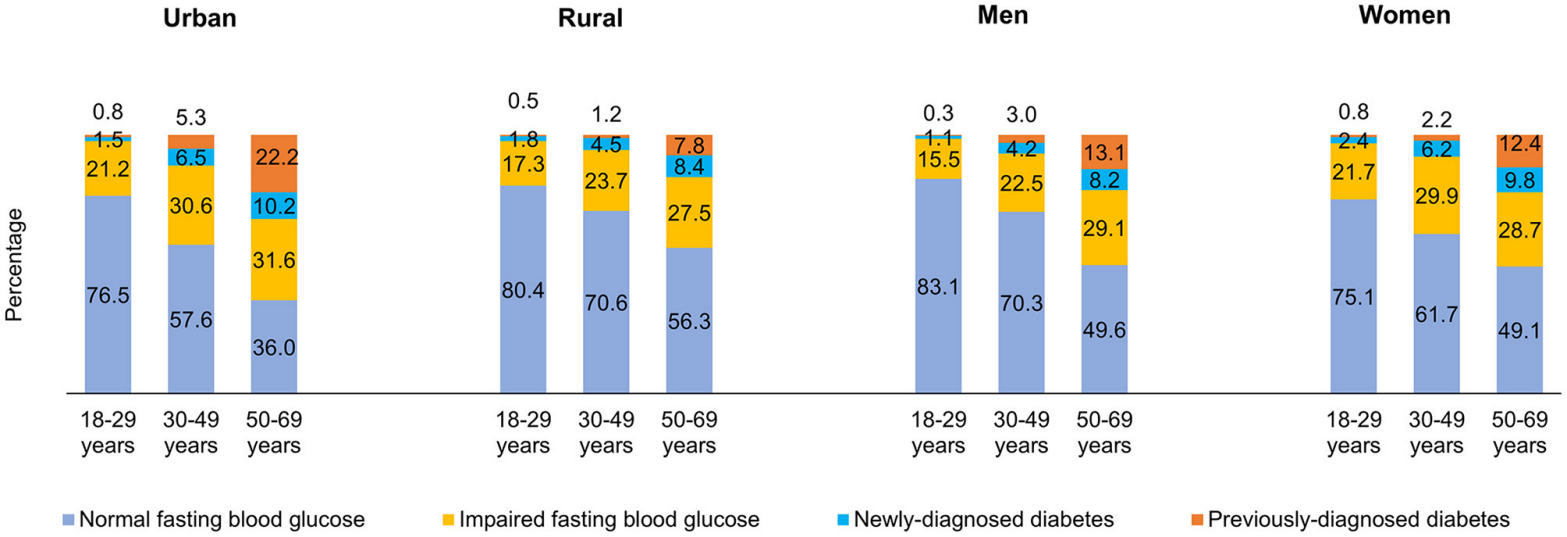
Distribution (Percentage) of fasting blood glucose among adults by area of residence, sex and age categories.

**Figure 3:**
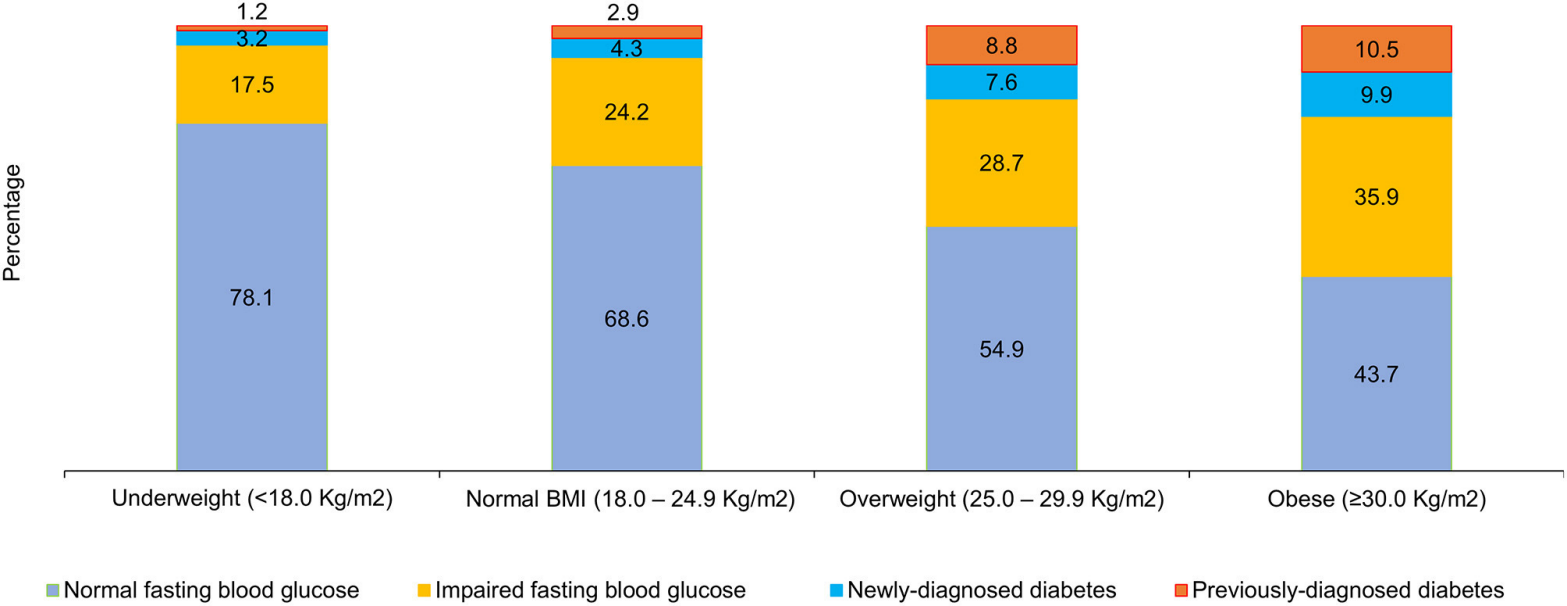
Distribution of fasting blood glucose among adults (18–69 years) by BMI (Percentage).

Figure 2 shows the distribution of adults by FBG values across different age categories. The prevalence of IFG (31.6%), previously-diagnosed by a doctor or health professional with diabetes (22.2%) and newly-diagnosed with raised FBG levels during the survey (10.2%) was highest among urban adults aged 50–69 years. The highest proportion of younger men had normal FBG levels (83.1%) (Figure 2). The prevalence of IFG was higher with increasing BMI. A higher proportion of adults with raised FBG levels and those previously-diagnosed belonged to the obese BMI category of ≥30.0 Kg/m^2^. Nearly a quarter proportion of the adults with normal BMI had IFG, 2.9% were previously-diagnosed and 4.3% were newly-diagnosed in the survey (Figure 3).

The prevalence of raised FBG was 9.3% and it was highest in adults aged 50–69 years (21.8%), those obese (20.5%), overweight (16.4%) and from urban areas (14.3%) (Table 2). The *p*-value across age groups and BMI categories were statistically significant (*p* < 0.001).

**Table 2:** Determinants (adjusted OR) of awareness and prevalence of diabetes among adults aged 18–69 years in India.

**Variables**	***N*** **(weighted)**	**Awareness**				**Prevalence**			
		**Univariate analysis**		**Multivariable logistic regression**		**Univariate analysis**		**Multivariable logistic regression**	
		***n* [%]**	*p* value^*^	**OR (95% CI)**	***p* value**	***n* [%]**	*p* value^*^	**OR (95% CI)**	***p* value**
**Residence**									
Urban	3,137	258 [8.2]	**<0.001**	1	**<0.001**	450 [14.3]	**<0.001**	1	**<0.001**
Rural	6,584	156 [2.4]		2.04 (1.61–2.59)		454 [6.9]		1.47 (1.25–1.73)	
**Sex**									
Men	5,036	218 [4.3]	**0.003**	1	0.119	427 [8.5]	**0.004**	1	0.146
Women	4,685	196 [4.2]		0.82 (0.63–1.05)		477 [10.2]		1.14 (0.96–1.36)	
**Age groups**									
18–29 years	2,853	14 [0.5]	**<0.001**	1		64 [2.2]	**<0.001**	1	
30–49 years	4,681	121 [2.6]		3.10 (1.78–5.40)	**<0.001**	364 [7.8]		2.77 (2.09–3.67)	**<0.001**
50–69 years	2,187	279 [12.8]		15.77 (9.11–27.27)	**<0.001**	476 [21.8]		8.89 (6.66–11.87)	**<0.001**
**Education status**									
Received education	6,847	319 [4.7]	**0.001**	1	**0.007**	649 [9.5]	0.338	1	**0.002**
No education	2,874	95 [3.3]		0.68 (0.51–0.90)		255 [8.9]		0.75 (0.63–0.90)	
**BEHAVIORAL RISK FACTORS**									
**Current tobacco use (any form)**									
No	6,513	325 [5.0]	0.015	1	**0.003**	675 [10.4]	**<0.001**	1	**0.012**
Yes	3,208	89 [2.8]		1.56 (1.17–2.08)		229 [7.1]		1.27 (1.05–1.54)	
**Current alcohol use**									
No	8,164	354 [4.3]	0.989	1	0.148	773 [9.5]	0.189	1	0.057
Yes	1,557	60 [3.9]		1.29 (0.91–1.82)		131 [8.4]		1.25 (0.99–1.58)	
**Physical activity**									
Sufficient	5,719	173 [3.0]	**<0.001**	1	0.112	441 [7.7]	**<0.001**	1	0.346
Insufficient	4,002	241 [6.0]		1.20 (0.96–1.50)		463 [11.6]		1.08 (0.92–1.26)	
**METABOLIC RISK FACTORS**									
**BMI**									
Normal	5,198	151 [2.9]	**<0.001**	1		373 [7.2]	**<0.001**	1	
Underweight	1,863	23 [1.2]		0.75 (0.47–1.21)	0.242	82 [4.4]		0.81 (0.63–1.05)	0.118
Overweight	1,895	166 [8.8]		1.32 (0.98–1.76)	0.065	311 [16.4]		1.45 (1.18–1.79)	**<0.001**
Obesity	590	62 [10.5]		1.37 (0.93–2.01)	0.108	121 [20.5]		1.65 (1.25–2.18)	**<0.001**
**Central obesity**									
No	6,492	122 [1.9]	**<0.001**	1	**<0.001**	366 [5.6]	**<0.001**	1	**<0.001**
Yes	3,071	282 [9.2]		2.03 (1.49–2.77)		522 [17.0]		1.48 (1.20–1.82)	
**Raised blood pressure**									
No	6,917	156 [2.3]	**<0.001**	1	**<0.001**	451 [6.5]	**<0.001**	1	**<0.001**
Yes	2,783	258 [9.3]		1.99 (1.58–2.51)		453 [16.3]		1.48 (1.27–1.73)	
**Reported raised cholesterol**									
No	9,592	367 [3.8]	**<0.001**	1	**<0.001**	847 [8.8]	**<0.001**	1	**<0.001**
Yes	129	47 [36.4]		3.85 (2.50–5.91)		57 [44.2]		2.80 (1.90–4.13)	

* *Chi-square test. P-value < 0.05 is considered statistically significant*.

Among those with diabetes (9.3%), nearly half were aware (45.8%), more than one-third were on treatment (36.1%), and only 15.7% had their blood glucose levels under control (Figure 1). Significantly higher proportions of adults who were aware and on treatment were older adults, urban residents, men, with metabolic risk factors and who received any level of education. While the control levels were higher in women, rural adults and those without any education though not statistically significant (Tables 2, 3). Awareness and treatment levels were statistically significant for among those with high BMI (*p*-value < 0.001) (Tables 2, 3).

**Table 3:** Determinants (adjusted OR) of treatment and control of diabetes among adults aware of diabetes aged 18–69 years in India.

**Variables**	***N*** **(weighted)**	**Treatment**				**Control**			
		**Univariate analysis**		**Multivariable logistic regression**		**Univariate analysis**		**Multivariable logistic regression**	
		***n* [%]**	*p* value^*^	**OR (95% CI)**	***p* value**	***n* [%]**	*p* value^*^	**OR (95% CI)**	***p* value**
**Residence**									
Urban	258	212 [82.2]	**0.023**	1	0.481	83 [32.2]	0.260	1	
Rural	156	114 [73.1]		1.24 (0.68–2.28)		59 [37.8]		1.12 (0.68–1.86)	0.653
**Sex**									
Men	218	180 [82.6]	0.074	1		69 [31.7]	0.387	1	
Women	196	146 [74.5]		0.51 (0.25–1.04)	0.063	73 [37.2]		1.18 (0.67–2.05)	0.568
**Age groups**									
18–29 years	14	3 [21.4]	**<0.001**	1		11 [78.6]	0.110	1	
30–49 years	121	86 [71.1]		3.79 (0.90–15.89)	0.069	36 [29.8]		0.12 (0.03–0.49)	**0.003**
50–69 years	279	237 [84.9]		8.34 (2.03–34.22)	**0.003**	95 [34.1]		0.14 (0.04–0.53)	**0.004**
**Education status**									
Received education	319	255 [79.9]	0.277	1		103 [32.3]	0.170	1	
No education	95	71 [74.7]		0.94 (0.45–1.97)	0.868	39 [41.1]		1.53 (0.83–2.83)	0.171
**BEHAVIORAL RISK FACTORS**									
**Current tobacco use (any form)**									
No	325	252 [77.5]	0.252	1		115 [35.4]	0.317	1	
Yes	89	74 [83.1]		0.83 (0.38–1.82)	0.635	27 [30.3]		1.49 (0.80–2.79)	0.208
**Current Alcohol use**									
No	354	282 [79.7]	0.398	1		115 [32.5]	0.077	1	
Yes	60	44 [73.3]		0.47 (0.19–1.16)	0.102	27 [45.0]		2.71 (1.33–5.53)	**0.006**
**Physical activity**									
Sufficient	173	121 [69.9]	**<0.001**	1		49 [28.3]	**0.019**	1	
Insufficient	241	205 [85.1]		1.79 (1.02–3.14)	**0.042**	93 [38.6]		1.94 (1.21–3.11)	**0.006**
**METABOLIC RISK FACTORS**									
**BMI**									
Normal	151	118 [78.1]	**0.002**	1		50 [33.1]	0.133	1	
Underweight	23	11 [47.8]		0.40 (0.13–1.20)	0.102	12 [52.2]		1.11 (0.39–3.16)	0.845
Overweight	166	132 [79.5]		0.71 (0.32–1.58)	0.405	60 [36.1]		1.24 (0.68–2.27)	0.490
Obesity	62	53 [85.5]		1.25 (0.46–3.40)	0.661	16 [25.8]		0.75 (0.34–1.66)	0.480
**Central obesity**									
No	122	84 [68.9]	**0.003**	1		48 [39.3]	0.136	1	
Yes	282	232 [82.3]		1.89 (0.85–4.22)	0.119	91 [32.3]		0.67 (0.35–1.27)	0.215
**Raised blood pressure**									
No	156	105 [67.3]	**<0.001**	1		53 [34.0]	0.757	1	
Yes	258	221 [85.7]		2.40 (1.38–4.17)	**0.002**	89 [34.5]		0.95 (0.59–1.52)	0.822
**Reported Raised cholesterol**									
No	367	281 [76.6]	**0.002**	1		127 [34.6]	0.982	1	
Yes	47	45 [95.7]		4.25 (0.92–19.63)	0.064	15 [31.9]		0.92 (0.45–1.86)	0.809

* *Chi-square test. P value < 0.05 is considered statistically significant*.

In the multivariate analysis, older age, metabolic risk factors namely overweight, obesity, central obesity, raised blood pressure and raised cholesterol were all significantly associated with an increased risk of diabetes (*p* < 0.001). Adults aged 50–69 years had more than 8.89 times higher odds of diabetes. Low physical activity and alcohol use showed risk but were not statistically significant (Table 2). Awareness levels of diabetes were 15.77 times higher among adults aged 50–69 years and 3.10 times among 30–49 years. These findings were statistically significant (*p* < 0.001). Adults who reported raised cholesterol had 3.85 times the odds of being aware of diabetes status, while those with central obesity (OR: 2.03) and hypertension (OR: 1.99) were of two times higher odds of being aware. These odds were statistically significant (*p* < 0.001) (Table 2). Older adults had higher odds of being on treatment for diabetes among those aware, while the control status was better among younger adults. Similar to awareness, those with increased BMI, central obesity, raised blood pressure and known raised cholesterol were on treatment, though the findings were not statistically significant (Table 3).

The majority of the urban adults sought both consultation and treatment from practitioners of the allopathic system of medicine (79.1%). A nearly similar proportion of adults (18–69 years) of their education and area of residence status sought consultation (84.0%) and treatment (78.8%) from allopathic practitioners. Rural residents in a higher proportion had taken consultation (24.6%) and treatment (18.2%) from practitioners of traditional systems of medicine (AYUSH) than urban residents (Supplementary Table S2).

Among the surveyed health facilities, more than 90% of public secondary health care facilities provided screening, laboratory and management services for diabetes. While, among the public primary care facilities, screening and management services for diabetes were available in 81.9% and 93.7% of facilities, respectively. Counseling services were available only in one-quarter of public primary (25.1%) and a half (50.8%) of the secondary care facilities (Supplementary Table S3).

## Discussion

The prevalence of diabetes and impaired glucose tolerance has been estimated to be 9.3% and 24.5%, respectively based on the nationally representative sample of adults aged 18–69-years in the National NCD Monitoring Survey. These findings highlight the impending burden of diabetes especially given the high population base and demographic transition in India. The survey also points out that almost half of diabetics are unaware of their raised fasting glucose status and that early diagnosis and treatment are primary for preventing complications, ensuring longevity and better quality of life. Across the globe, 10.4% of the population from high-income countries, 9.5% from middle-income and 4.0% from low-income countries were diabetic in 2019 (8). The South-East-Asia-Region ranked third in the prevalence of diabetes in 2019 with India ranking second to China (8). The prevalence of diabetes is projected to rise by the year 2045, with a nearly equal proportion from high-income (11.9%) and middle-income (11.8%) countries; and 4.7% in low-income countries (8). Few other recent epidemiological surveys, showed the prevalence of DM in India ranged from 5 to 17% (13–16). This paper findings identify groups that are at a specific disadvantage, highlighting the need for improving access to both preventive and curative health care among these groups. It also provides empirical evidence for policy formulation in the area of NCDs, especially would call for actions to prevent the occurrence of disease as well as to improve the reach of health systems for diabetes care. The study recommends robust data management under the National Program for Control and Prevention of Cancer, Diabetes, Cardiovascular Diseases and Stroke (NPCDCS) for both public and private health facilities. Also, a need for community-based implementation strategies for treatment and control like strengthening counseling services through grassroot health workers like the ASHA, either incentivise or disincentivise schemes for increasing physical activity or reduction of obesity.

This survey reports that the prevalence of DM was two times higher in urban areas (14.3%) than in rural areas (6.9%). Urban areas also showed a high prevalence of IFG. These findings are more robust as the NNMS has the advantages of a national sample equally distributed among both urban and rural areas. Several large epidemiological studies in India have reported similar findings (16–19). The ICMR-INDIAB study reported the prevalence of diabetes in urban areas being higher than rural areas, being highest in the age group of 55–64 years (Urban: nearly 25% and rural areas: nearly 10%) (16). Urban predominance of diabetes is an influence of a multitude of factors like rapid urbanization, the prevalence of overweight and obesity in consequence of inactive lifestyle and changing dietary habits (1, 8, 16–19). But the proportions in rural areas are also worrisome, with an equally high prevalence of IFG and raised FBG, reflecting the expanding urbanization. Gupta et al. 2020, discussed the reduction of the conventional rural-urban differences in the prevalence of DM (20). Their study findings on diabetes prevalence in rural areas was similar to urban studies by Goswami et al. in 2016, and Singh et al. in 2012 undertaken in the same geographic locations in India (20–22). This urban-rural narrowing has been reported across the globe (8, 23). The International Diabetes Federation reported, 67.0% of adults living with diabetes across the world were urban residents, but also notified the rising prevalence of DM in the rural areas (10.8%–Urban vs. 7.2%–Rural) (8). Thus, the emerging challenges with DM in rural India cannot be overlooked, rapid mechanisms are needed to prevent and halt the rise.

The prevalence of IFG was higher than diabetes, specifically most affected were aged 50–69 years, followed by those aged 30–49 years and 18–29 years which constitute to be the most productive years of life. The World Health Organization–IDF reported the 35–64 year age group to be the most prevalent group with diabetes in the developing countries compared to the 65+ years group in the developed countries (2). It is projected that by 2030, this age-wise burden shall only increase with no alteration in its course (2). Thus, amplified efforts of screening, early interventions, awareness and health promotion among the younger adults would help prevent and halt the progression rates from IFG to DM. These findings from our national survey are imperative for aggressive policy planning and action.

The current study showed, the prevalence of IFG and DM being pre-dominant among women. This could be influenced by the sex-related differences in lifestyle and risk factors (1, 16, 18). Women are more likely to be with higher BMI (obese or overweight) than men and thus be expected to have a higher prevalence of DM (1, 16). Both IFG and DM were more prevalent among adults with metabolic risk factors—overweight, obesity, central obesity than those with normal BMI or underweight (16–19, 24). Also, 44.2% with raised cholesterol and 16.3% with hypertension had diabetes thus revealing the cardio-metabolic and co-morbid behavior of DM. As these co-morbid factors share common risk factors, adpating lifestyle alterations like weight management, sufficient physical activity, consumption of adequate servings of fruits and vegetables and other dietary modifications can together benefit their prevention and control (25).

Notably, awareness, treatment and control of DM were better among older adults, men, urban residents and those who received some education. Those with raised cholesterol and raised blood pressure had better awareness levels, were taking anti-diabetic medication and had their blood glucose under control. This could be explained by improved awareness on DM in urban areas; better access and affordability to care; routine screening of blood glucose along other co-morbid factors like blood pressure and cholesterol (1, 3, 16). Poor awareness and treatment levels in women can be attributed to poor access to treatment for women. Also, being educated enables one to understand and be willing to adopt healthy behaviors. A high proportion of adults previously-diagnosed vs. newly-diagnosed DM in both urban and rural areas as well as among older adults, highlights the sustained efforts by the Government of India through the NPCDCS program (26). However, still a large proportion remains undiagnosed and are not adherent to treatment and this is a problem in the low-and-middle-income countries like India (8). Nearly 70% of primary and more than 90% of secondary public health facilities surveyed provided screening, laboratory, and management services for diabetes, but majorly lacked counseling services (public primary−25% and public secondary−51%). Thus, emphasizing the need to expand and strengthen the initiative especially at the primary care level in both urban and rural areas. Also, stronger actions are needed to identify the younger adults and women who are more likely to be missed from diagnosis and or treatment.

More than three-fourth of adults in India sought care for diabetes from allopathic practitioners and more than 10% from AYUSH practitioners and this proportion was higher among rural residents (18.2–24.6% from AYUSH) and older adults. Indicating that the rural residents and older adults are more oriented and receptive to the Indian traditional systems of medicine. This provides a new dimension to policymakers in promoting AYUSH services for preventive and early diabetes care. Also, encouraging and expanding traditional medicine services in urban areas as well as creating awareness among younger adults are better alternatives to lessen the current and future burden on health systems. Amalgamating allopathic and traditional systems as a holistic approach to diabetes and NCD care can help meet the rising burden on health systems (27, 28).

The strengths and limitations of this survey findings include the general strengths and limitations of NNMS that have been described elsewhere (10, 11). Several studies either provide random blood glucose estimates to report the prevalence of diabetes or self-reported history (17, 18, 21). We have used a combination of previously-diagnosed history and fasting blood glucose measurements to report diabetes prevalence in India. However, due to logistic reasons, we used capillary blood glucose for IFG and raised FBG estimations as a standard alternative to venous plasma blood glucose (12). Since it is a cross-sectional survey that recorded behavioral risk factors at the time of the survey and no baseline data, it is difficult to infer if some of these survey participants may have changed behavior after diagnosis of diabetes or possibly other chronic illnesses and therefore their relationship as causal factors are not significant. Our findings provide national estimates that can help inform policies to target populations at risk for diabetes based upon awareness, treatment and control levels. Also, provide a baseline to monitor the NCD targets under the global and national NCD framework to be achieved by 2025.

In conclusion, the level of morbidity and mortality from diabetes and its potential complications are enormous. Despite the presence, some of the persons continue to have behavioral risk factors and thus increasing their chances for complications. Producing periodic prevalence estimates, awareness, treatment and control levels as well as future projections for diabetes is essential to promote its prevention and encourage quality of care. Continuous monitoring and surveillance of diabetes as well as comprehensive health promotive and management interventions among diabetics are crucial in the progress of countries to achieve the WHO Global NCD Voluntary Targets by 2025.

## Data Availability Statement

The National Noncommunicable Disease Monitoring Survey (NNMS) report is available at: https://www.ncdirindia.org/nnms/. Further data are available upon request.

## Ethics Statement

The studies involving human participants were reviewed and approved by Institutional Ethics Committee of the Coordinating Center, Indian Council of Medical Research—National Centre for Disease Informatics and Research, Bengaluru, India. Every implementing agency obtained their ethics approval from their own Institutional Ethics Committee. The patients/participants provided their written informed consent to participate in this study. Approval no: NCDIR/IEC/2017/4 dated 03 Feb 2017.

## ICMR-NNMS Investigator, Co-Investigator and Collaborator Group

1. Prashant Mathur^†*^, Indian Council of Medical Research—National Center for Disease Informatics and Research, Bengaluru, Karnataka, India.
2. Vaitheeswaran Kulothungan^†^, Indian Council of Medical Research—National Center for Disease Informatics and Research, Bengaluru, Karnataka, India.
3. Sravya Leburu^†^, Indian Council of Medical Research—National Center for Disease Informatics and Research, Bengaluru, Karnataka, India.
4. Anand Krishnan, Center for Community Medicine, All India Institute of Medical Sciences, New Delhi, India.
5. Himanshu Kumar Chaturvedi, Indian Council Medical Research—National Institute of Medical Statistics, New Delhi, India.
6. Harshal Ramesh Salve, Center for Community Medicine, All India Institute of Medical Sciences, New Delhi, India.
7. Ritvik Amarchand, Center for Community Medicine, All India Institute of Medical Sciences, New Delhi, India.
8. Baridalyne Nongkynrih, Center for Community Medicine, All India Institute of Medical Sciences, New Delhi, India.
9. P. Ganesh Kumar, Indian Council Medical Research—National Institute of Epidemiology, Chennai, Tamil Nadu, India.
10. Vinay Urs K. S., Indian Council of Medical Research—National Center for Disease Informatics and Research, Bengaluru, Karnataka, India.
11. A. Laxmaiah, Division of Community Studies, Indian Council of Medical Research–National Institute of Nutrition, Hyderabad, Telangana, India.
12. Manjit Boruah, Department of Community Medicine, Assam Medical College, Dibrugarh, Assam, India.
13. Sanjeev Kumar, Department of Community and Family Medicine, All India Institute of Medical Sciences, Bhopal, Madhya Pradesh, India.
14. Binod Kumar Patro, Department of Community and Family Medicine, All India Institute of Medical Sciences, Bhubaneshwar, Odisha, India.
15. Pankaja Ravi Raghav, Department of Community Medicine and Family Medicine, All India Institute of Medical Sciences, Jodhpur, Rajasthan, India.
16. Prabu Rajkumar, Indian Council of Medical Research—National Institute of Epidemiology, Chennai, Tamil Nadu, India.
17. P. Sankara Sarma, Achutha Menon Center for Health Science Studies, Sree Chitra Tirunal Institute for Medical Sciences and Technology, Thiruvananthapuram, Kerala, India.
18. Rinku Sharma, Center for Noncommunicable Diseases, National Center for Disease Control, Directorate General of Health Services, New Delhi, India.
19. Muralidhar Tambe, Department of Community Medicine, B J Govt. Medical College, Pune, Maharashtra, India.
20. K. R. Thankappan, Department of Public Health and Community Medicine, Central University Kerala, Kasaragod, Kerala, India.
21. N. Arlappa, Division of Community Studies, Indian Council of Medical Research–National Institute of Nutrition, Hyderabad, Telangana, India.
22. Tulika Goswami Mahanta, Department of Community Medicine/Prevention and Social Medicine, Tezpur Medical College, Tezpur, Assam, India.
23. Rajnish P. Joshi, Department of General Medicine, All India Institute of Medical Sciences, Bhopal, Madhya Pradesh, India.
24. Neeti Rustagi, Department of Community Medicine and Family Medicine, All India Institute of Medical Sciences, Jodhpur, Rajasthan, India.
25. Sonia Gupta, Center for Noncommunicable Diseases, National Center for Disease Control, Directorate General of Health Services, New Delhi, India.
26. Binod Kumar Behera, Department of Community and Family Medicine, All India Institute of Medical Sciences, Bhubaneshwar, Odisha, India.
27. Sangita Chandrakant Shelke, Department of Community Medicine, B J Govt. Medical College, Pune, Maharashtra, India.
28. Abhiruchi Galhotra, Department of Community and Family Medicine, All India Institute of Medical Sciences, Raipur, Chattisgarh, India.
29. Pranab Jyoti Bhuyan, Regional Director Office, Ministry of Health and Family Welfare, Guwahati, Assam, India.
30. Abhijit P. Pakhare, Department of Community and Family Medicine, All India Institute of Medical Sciences, Bhopal, Madhya Pradesh, India.
31. Dewesh Kumar, Department of preventive and social medicine, Rajendra Institute of Medical Sciences, Ranchi, Jharkhand, India.
32. Roshan K. Topno, Department of Epidemiology, Indian Council of Medical Research—Rajendra Memorial Research Institute of Medical Sciences, Patna, Bihar, India.
33. Manoj Kumar Gupta, Department of Community Medicine and Family Medicine, All India Institute of Medical Sciences, Jodhpur, Rajasthan, India.
34. Atulkumar V. Trivedi, Department of Community Medicine, Government Medical College, Bhavnagar, Gujarat, India.
35. Suneela Garg, Department of Community Medicine, Maulana Azad Medical College and Associated Hospitals, New Delhi, India.

## Author Contributions

SL, VK, and PM contributed to the concept, design of the paper, and involved in revision of the manuscript. VK and SL developed the analysis plan. VK was involved in data management and statistical analyses. SL drafted the manuscript with expert review and inputs from PM and VK. PM reviewed the plan, received funding for the study, and was the principal investigator. All authors were part of the central coordinating unit primarily involved in investigation and approved the final version of the manuscript.

## Funding

The Ministry of Health and Family Welfare (MoHFW), Government of India, funded this survey. (Dy.No.C-707, dated 06 July 2015). The funders only provided the funds and had no role in the study planning, implementation and preparation of the manuscript.

## Conflict of Interest

The authors declare that the research was conducted in the absence of any commercial or financial relationships that could be construed as a potential conflict of interest.

## Publisher's Note

All claims expressed in this article are solely those of the authors and do not necessarily represent those of their affiliated organizations, or those of the publisher, the editors and the reviewers. Any product that may be evaluated in this article, or claim that may be made by its manufacturer, is not guaranteed or endorsed by the publisher.
