# Supplementary material for: Prevalence, Awareness, Treatment and Control of Diabetes in India From the Countrywide National NCD Monitoring Survey
Source: Front Public Health. 2022 Mar 14;10:748157. doi: 10.3389/fpubh.2022.748157 (PMC8964146; doi:10.3389/fpubh.2022.748157)
Supplement: Supplementary file 1 [file Table_1.docx]

**Supplementary Files**

**Supplementary Table S1: Mean fasting blood glucose levels (mg/dl) among adults 18-69 years**

| **Variables** | **Mean fasting blood glucose (mg/dl)** | | |
| --- | --- | --- | --- |
|  | **n (weighted)*** | **Mean (SD)** | **p-value** |
| **Residence** | | |  |
| Urban | 3114 | 101.57 (31.3) | <0.0001 |
| Rural | 6570 | 94.41 (23.65) |  |
| **Sex** | | |  |
| Men | 5012 | 95.07 (27.35) | <0.0001 |
| Women | 4672 | 98.48 (26.40) |  |
| **Age groups** | | |  |
| 18-29 years | 2853 | 89.57 (15.85) | <0.0001 |
| 30-49 years | 4663 | 96.09 (24.24) |  |
| 50-69 years | 2168 | 107.47 (38.30) |  |
| Overall (18-69 years) | 9684 | 96.7 (26.95) |  |
| n (weighted): no. of participants; SD: Standard deviation  *Adults who were previously diagnosed with diabetes and did not participate in the fasting blood glucose measurement are excluded. | | | |

**Supplementary Table S2: Diabetes care-seeking patterns among adults 18-69 years in India**

| **Variables** | **Consultation** | | | | **Seeking Treatment** | | | | **Consultation and Treatment** | | | |
| --- | --- | --- | --- | --- | --- | --- | --- | --- | --- | --- | --- | --- |
|  | **Allopathy** | | **AYUSH*** | | **Allopathy** | | **AYUSH*** | | **Allopathy** | | **AYUSH*** | |
|  | **n** | **%** | **n** | **%** | **n** | **%** | **n** | **%** | **n** | **%** | **n** | **%** |
| **Residence** | | | | | | | | | | | | |
| Urban | 221 | 85.6 | 37 | 14.3 | 212 | 82.4 | 28 | 10.7 | 204 | 79.1 | 28 | 10.7 |
| Rural | 127 | 81.4 | 38 | 24.6 | 114 | 72.8 | 28 | 18.2 | 102 | 65.1 | 28 | 18.2 |
| **Sex** | | | | | | | | |  |  |  |  |
| Men | 183 | 83.8 | 40 | 18.1 | 179 | 82.2 | 28 | 12.9 | 166 | 75.8 | 28 | 12.9 |
| Women | 165 | 84.2 | 35 | 18.2 | 147 | 75.0 | 28 | 14.3 | 140 | 71.6 | 28 | 14.3 |
| **Age groups** | | | | | | | | | | | | |
| 18-29 years | 6 | 43.1 | 2 | 15.9 | 3 | 20.9 | 0 | 0.0 | 3 | 20.9 | 0 | 0.0 |
| 30-49 years | 96 | 79.1 | 21 | 17.0 | 86 | 71.2 | 14 | 11.5 | 83 | 68.9 | 14 | 11.5 |
| 50-69 years | 246 | 88.3 | 52 | 18.8 | 237 | 85.1 | 42 | 15.2 | 220 | 78.8 | 42 | 15.2 |
| Overall (18-69 years) | 348 | 84.0 | 75 | 18.1 | 326 | 78.8 | 56 | 13.6 | 306 | 73.8 | 56 | 13.6 |
| **Education status** | | | | | | | | | | | | |
| Received education | 267 | 83.7 | 58 | 18.1 | 255 | 79.9 | 43 | 13.5 | 240 | 74.8 | 43 | 13.5 |
| No education | 81 | 85.0 | 17 | 18.3 | 71 | 74.9 | 13 | 13.9 | 66 | 70.5 | 13 | 13.9 |
| *AYUSH – Alternative systems of medicine that include Ayurveda, Yoga and naturopathy, Unani, Siddha and Homeopathy. | | | | | | | | | | | | |

**Supplementary Table S3: Proportion of primary and secondary public health facilities providing services for diabetes care**

| **Availability of services for Diabetes** | **Public primary care facilities** | | **Public secondary facilities (CHC/DH)*** | |
| --- | --- | --- | --- | --- |
|  | **n** | **%** | **n** | **%** |
| **Screening** | 440 | 81.9 (78.4-85.0) | 696 | 92.8 (90.7-94.4) |
| **Laboratory** | 372 | 69.3 (65.2-73.0) | 724 | 96.5 (95.0-97.6) |
| **Management** | 503 | 93.7 (91.3-95.4) | 746 | 99.5 (98.6-99.8) |
| **Counselling** | 135 | 25.1 (21.6-29.0) | 381 | 50.8 (47.2-54.4) |
| *CHC-Community Health Centre and DH – District Hospital | | | | |
